# A rock physics modelling algorithm for simulating the elastic parameters of shale using well logging data

**DOI:** 10.1038/s41598-018-29755-2

**Published:** 2018-08-14

**Authors:** Bing Wang, Yurong Chen, Jing Lu, Wujun Jin

**Affiliations:** 1 State Key Laboratory of Petroleum Resources and Prospecting, China University of Petroleum, Beijing, 102249 China; 2 Key Laboratory of Earth Prospecting and Information Technology, Beijing, 102249 China; 3 Petroleum Exploration and Production Research Institute, SINOPEC, Beijing, 100083 China

## Abstract

As a high-resolution geophysical method employed by the oil and gas industry, well logging can be used to accurately investigate reservoirs. Challenges associated with shale gas reservoir exploration increase the importance of applying elastic parameters or velocity at the logging scale. An efficient shale rock physics model is the foundation for the successful application of this method. We propose a procedure for modelling shale rock physics in which an appropriate modelling method is applied for different compositions of shale rock. The stiffnesses of the kerogen and fluid (oil, gas or water) mixture are obtained with the Kuster-Toksöz model, which assumes that the fluid is included in the kerogen matrix. A self-consistent approximation method is used to model clay, where the clay pores are filled with formation water. The Backus averaging model is then used to simulate the influence of laminated clay and laminated kerogen. Elastic parameter simulations using well logging data show the importance of treating the volume fractions of laminated clay and kerogen carefully. A comparison of the measured compressional slowness and modelled compressional slowness shows the efficiency of the proposed modelling procedure.

## Introduction

Rock physics concepts are widely used to establish connections between rock compositions and macroscopic properties. A proper rock physics model can help effectively study the rock characteristics. As an unconventional resource, shale gas has recently received increasing attention. Many challenges have been observed in shale gas reservoir exploration and exploitation. The development of an efficient shale rock physics model is important.

At present, rock physics models of shale are mainly aimed at seismic rock physics modelling. Early research on shale rock physics models was performed by Vernik and Nur^1^, and the efficiency of such models was first investigated in Bakken shale^1,2^. Hornby *et al*. used the self-consistent approximation (SCA) method and differential effective medium (DEM) method to study the complicated mineralogical content and distribution of pores and microcracks in shale^3^. Carcione introduced the viscoelastic anisotropic theory to study the elastic attenuation of shale^4^. The complicated microstructure of organic-rich shale can be modelled using the Backus average theory^5^. Carcione *et al*. compared the Gassmann and Backus average theories for fluid saturation in source rocks^4,6^. The Gassmann theory is more general than the Backus average method because it does not make any assumptions on the shape of the pores and grains^7^. The Gassmann method only works well for low frequencies in seismic data (<100 Hz)^8^. Using the SCA and Backus average method, Guo *et al*. constructed rock physics templates for shale and studied the relation among mineralogy, porosity, brittleness, and the elastic parameters of rocks and their corresponding seismic responses^9^. Effects of laminated clay and kerogen can be studied using the Backus averaging method. As an inclusion model, the Kuster-Toksöz model describes various pore geometries using different pore aspect ratios, which can well characterize the actual storage spaces in reservoir. This model is the most commonly used rock physics model in exploration geophysics, although certain limitations apply. The Kuster-Toksöz model assumes idealized ellipsoidal inclusion shapes and is suitable for rocks with low porosity and low fracture density^8^.

The target of this study is to find a shale rock physics modelling procedure towards sonic logging (≈10^4^ HZ) to establish the relationship between shale elastic properties, e.g., the porosity, and shale composition, e.g., matrix mineral types, clay content, kerogen content, layered clay content and layered kerogen content. By using different modelling methods for different compositions of shale rock, the elastic modulus of the shale can be simulated. Due to the layered clay and layered kerogen, shale rock presents as a transverse isotropic material, which should be treated carefully. Application on real well logging data shows the efficiency of our modelling method.

## Theories of Rock Physics Models

### SCA model

Berryman presents a general form of SCAs for N-phase composites^10^:

1 $$\sum _{{\rm{i}}=1}^{{\rm{n}}}{{\rm{x}}}_{i}({K}_{i}-{K^{\prime} }_{SC}){\beta }^{^{\prime} i}=0$$

2 $$\sum _{{\rm{i}}=1}^{{\rm{n}}}{{\rm{x}}}_{i}({\mu }_{i}-{\mu ^{\prime} }_{SC}){\zeta }^{^{\prime} i}=0$$

where i refers to the i*th* material; x_*i*_ is its volume fraction; *K*_i_ and *μ*_i_ are the bulk and shear moduli for each fraction, respectively; and ${K^{\prime} }_{{\rm{s}}c}$ and ${\mu ^{\prime} }_{{\rm{s}}c}$ are the effective bulk and shear moduli, respectively. The values of *β*^*i*^ and *ζ*^i^ are coefficients for different-shaped cracks. The summation is performed over all phases, including minerals and pores.

We consider that the factors ${\beta }^{^{\prime} {\rm{i}}}$ and ${\zeta }^{^{\prime} {\rm{i}}}$, which describe the geometry of an inclusion made of phase *i* within a background medium (denoted with subscript m) when the aspect ratio *α* ≤ 1, is given as follows:

3 $${\beta }^{^{\prime} i}=\frac{{K}_{m}+\frac{4}{3}{\mu }_{i}}{{K}_{i}+\frac{4}{3}{\mu }_{i}+\pi \alpha {\mu }_{m}\frac{3{K}_{m}+{\mu }_{m}}{3{K}_{m}+4{\mu }_{m}}}$$

4 $${\zeta }^{\text{'}i}=\frac{1}{5}[\frac{8{\mu }_{m}}{4{\mu }_{i}+\pi \alpha {\mu }_{m}(1+2\frac{3{K}_{m}+{\mu }_{m}}{3{K}_{m}+4{\mu }_{m}})}+\frac{{K}_{i}+\frac{2}{3}({\mu }_{i}+{\mu }_{m})}{{K}_{i}+\frac{4}{3}{\mu }_{i}+\pi \alpha {\mu }_{m}\frac{3{K}_{m}+{\mu }_{m}}{3{K}_{m}+4{\mu }_{m}}}]$$

where *K*_m_ and *μ*_m_ are the bulk and shear moduli for a matrix, respectively.

The limitation of this model is that the porosity is assumed to be incompatible (it prevents hydraulic communication and pore pressure equilibrium) and the wavelength is much larger than the size of the inclusion. This method is suitable for moderate porosity. If the pore aspect ratio is relatively small, then this model must be used carefully. The equations above describe a high-frequency model, and the high frequencies do not allow sufficient time for pore pressures to balance.

If we wish to treat all constituents equally, the matrix material in the model is replaced with the effective material, which is similar to the case of porous solids with connected fluid and solid phases. In this work, the assumption of the SCA as a high-frequency model appears to be satisfied. Moreover, we can use the SCA model to calculate the effective moduli of the clay in which the bound water is contained as well as the effective moduli of all constituents.

### Kuster-Toksöz model

Kuster and Toksöz used the aspect ratio of pores to describe the pore shape. Based on the scattering theory, the relationship between the elastic modulus of the rock and the porosity or pore shape is established^11^. The stiffnesses of the mixture can be calculated using this model. If S is the fluid saturation *S* = Φ_*f*_/(Φ*f* + Φ_*k*_), then the stiffnesses can be expressed as follows:

5 $$\frac{{K}_{ke}}{{K}_{k}}=\frac{1+[4\mu k({K}_{f}-{K}_{k})/(3{K}_{f}+4{\mu }_{k}){K}_{k}]S}{1-[3({K}_{f}-{K}_{k})/(3{K}_{f}+4{\mu }_{k})]S}$$

and

6 $$\frac{{\mu }_{ke}}{{\mu }_{k}}=\frac{(1-S)(9{K}_{f}+8{\mu }_{k})}{9{K}_{k}+8{\mu }_{k}+S(6{K}_{k}+12{\mu }_{k})}$$

where Φ_*f*_ and Φ_*k*_ describe the volume of fluid contained in kerogen and the volume of kerogen, respectively; S describes the fluid saturation; *K*_*k*_ and ***μ***_*k*_ are the bulk and shear moduli of the kerogen, respectively; *K*_*ke*_ and ***μ***_*ke*_ are the effective bulk and shear moduli of the fluid contained in kerogen, respectively; and *K*_*f*_ is the bulk modulus for the fluid (mixtures of gas, oil and water). The density of the mixture is *ρ*_*if*_ = (Φ_*k*_*ρ*_*k*_ + Φ_*f*_*ρ*_*f*_)/(Φ_*k*_ + Φ_*f*_), where *ρ*_f_ and *ρ*_*k*_ refer to the density of the fluid contained in kerogen and the density of kerogen, respectively.

The Kuster-Toksöz expressions assume idealized ellipsoidal inclusion shapes. The equations above are formally limited to low porosity and low fracture density and describe a high-frequency model. In this work, we treat kerogen saturated with mixtures of gas, oil and water as inclusions in the KT model because kerogen particles as inclusions are approximately considered penny cracks. In this case, kerogen must be distributed randomly, and its effect is isotropic.

### Backus averaging method

Backus showed that in the long-wavelength limit, a stratified medium composed of layers of transversely isotropic materials (each with its symmetry axis normal to the strata) is also effectively anisotropic^12^, and he assumed that shale rock is a multiple-layer composite composed of laminated clay minerals and kerogen of lamination in texture, and a mixture of all other minerals in the work of Vernik and Nur^1^. The Backus averaging produces a transversely isotropic effective medium described by five stiffnesses *C*_*IJ*_, where:

7 $$\begin{array}{rcl}{C}_{11}^{\ast } & = & \langle {C}_{11}-{C}_{13}^{2}{C}_{33}^{-1}\rangle +{\langle {C}_{33}^{-1}\rangle }^{-1}{\langle {C}_{13}{C}_{33}^{-1}\rangle }^{2}\\ {C}_{33}^{\ast } & = & {\langle {{C}_{33}}^{-1}\rangle }^{-1}\\ {C}_{13}^{\ast } & = & {\langle {C}_{33}^{-1}\rangle }^{-1}\langle {C}_{13}{C}_{33}^{-1}\rangle \\ {C}_{55}^{\ast } & = & {\langle {C}_{55}^{-1}\rangle }^{-1}\\ {C}_{66}^{\ast } & = & \langle {C}_{66}\rangle \end{array}$$

where *C*_*IJ*_ represents the complex stiffnesses corresponding to the single constituents. The brackets 〈**·**〉 indicate the averages of the enclosed properties weighted by their volumetric proportions. According to our rock physics model, Backus averaging is defined as follows:

8 $$\langle \alpha \rangle ={f}_{m}{\alpha }_{m}+{f}_{c}{\alpha }_{c}+{f}_{k}{\alpha }_{k}$$

where *m* indicates a mixture of all other compositions, including quartz, calcite, dolomite and other rock minerals; *c* is the laminated clay mineral; *k* is the laminated kerogen; and *f* denotes the weight for averaging.

### Addressing the microcracks aspect ratio

Depending on the microstructures of rocks, a single- or multiple-aspect-ratio model is usually used to model the pore space^13^. In this work, we adopt a multiple-aspect-ratio algorithm. Each set of pores consists of a number of 100 aspect ratios, and the aspect ratio of each set of pore inclusions has a normal distribution, which is specified by an averaging value and a standard deviation. By this process, the modelling of the pore space can approximate the real case with increased accuracy.

### Shale elastic rock physics modelling workflows

**Figure 1:**
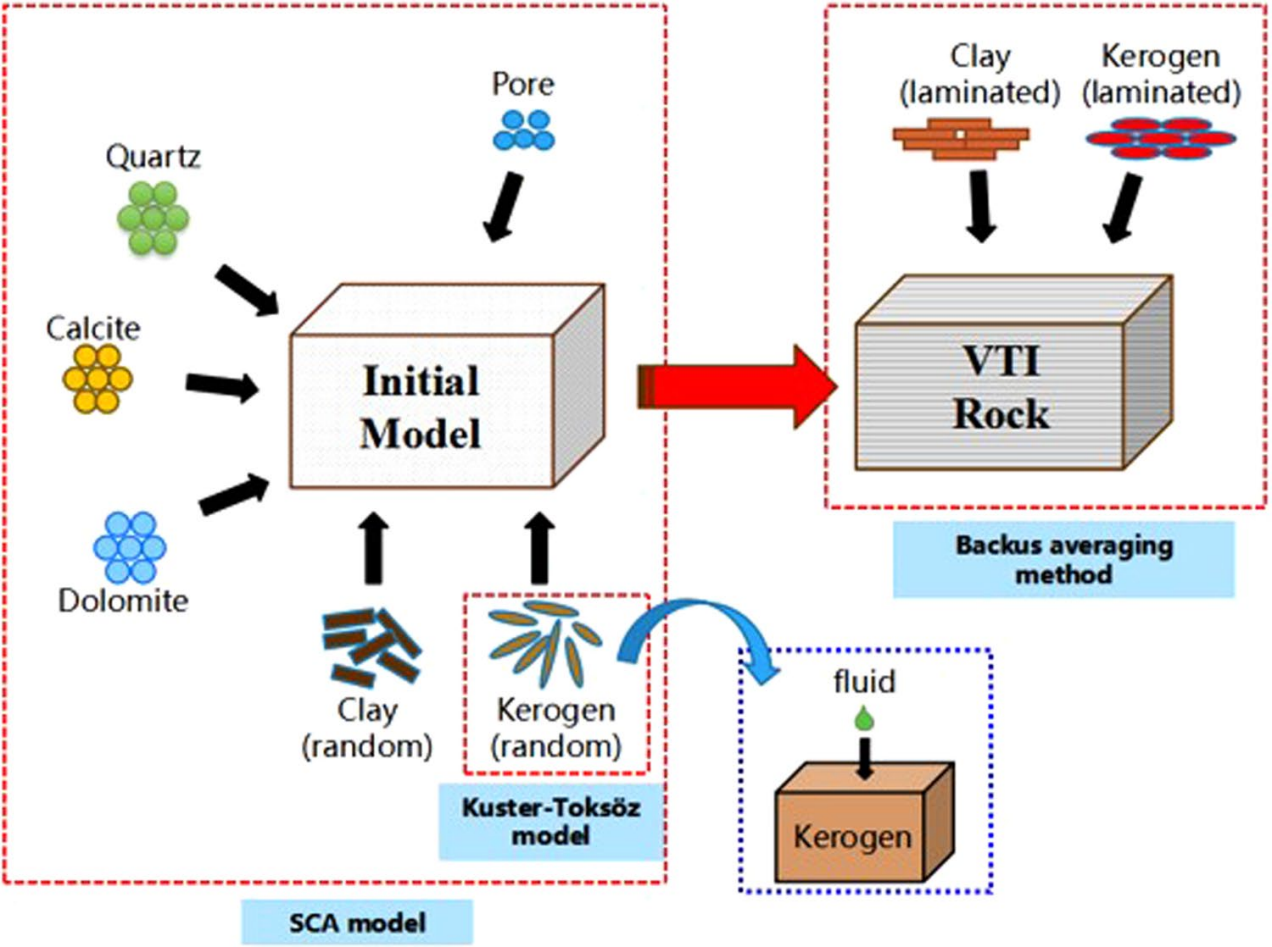
Schematic of the shale rock physics modelling procedure.

For high frequencies in acoustic logging measurements, we choose different modelling methods for different compositions of the shale. Here, we concentrate on modelling the elastic properties of shale. The modelling schematic is shown in Fig. 1.

The modelling steps are as follows:

1. The shale rock is divided into three parts: matrix (quartz, calcite and dolomite), clay and kerogen.
2. The effective elastic modulus of kerogen saturated with mixtures of gas, oil and water as inclusions is calculated by the Kuster-Toksöz model.
3. The effective bulk modulus and shear modulus of the clay fraction are calculated by the SCA (self-consistent) model, in which the bound water is contained in the clay. The formulas are as follows:
  $$\begin{array}{c}{f}_{bw}({K}_{w}-{K}_{C}^{\ast }){\beta }^{w}+{f}_{c}({K}_{c}-{K}_{C}^{\ast }){\beta }^{c}=0\\ {f}_{bw}({\mu }_{w}-{\mu }_{C}^{\ast }){\xi }^{w}+{f}_{c}({\mu }_{c}-{\mu }_{C}^{\ast }){\xi }^{c}=0\end{array}$$
  where *K*_*w*_ and ***μ***_*w*_ are the bulk and shear moduli of the fluid (water), respectively; *K*_*c*_ and ***μ***_*c*_ are the bulk and shear moduli of the clay, respectively; *α* is the crack aspect ratio, whose value is 1; and *f*_*bw*_ and *f*_*c*_ are the relative volumes of the clay-hydration water and clay, respectively (*f*_*bw*_ + *f*_*c*_ = 1).
4. According to the matrix mineral content, the skeleton part of the effective bulk and shear moduli can be calculated. The formula is as follows:
  $${K}_{me}=\sum _{i=1}^{i=N}{f}_{i}{K}_{i},\,{\mu }_{me}=\sum _{i=1}^{i=N}{f}_{i}{\mu }_{i}$$
  where *K*_*me*_ and ***μ***_*me*_ are the effective bulk and shear moduli of the matrix, respectively; and *K*_*i*_ and *μ*_*i*_ indicate the bulk and shear moduli for an inclusion of material i in skeleton minerals, respectively. Three species of matrix minerals are considered in this work: quartz, calcite and dolomite. Parameter *f*_*i*_ is the relative volume fraction of the *i*th type matrix mineral in the shale rock, and N is the total species of the matrix minerals.
5. Finally, the SCA (self-consistent) model is used to calculate the effective bulk and shear moduli of the whole shale formation, and it can obtain the isotropic effective elastic properties of shale.
  $$\begin{array}{c}{f}_{k}({K}_{ke}-{K}_{e}^{\ast }){\beta }^{k}+{f}_{cl}({K}_{C}^{\ast }-{K}_{e}^{\ast }){\beta }^{cl}+{f}_{m}({K}_{me}-{K}_{e}^{\ast }){\beta }^{m}=0\\ {f}_{k}({\mu }_{ke}-{\mu }_{e}^{\ast }){\xi }^{{\rm{k}}}+{f}_{cl}({\mu }_{C}^{\ast }-{\mu }_{e}^{\ast }){\xi }^{cl}+{f}_{m}({\mu }_{me}-{\mu }_{e}^{\ast }){\xi }^{m}=0\end{array}$$
  where *f*_*k*_, *f*_*cl*_ and *f*_*m*_ are the relative content of kerogen, clay and skeleton volume, respectively (*f*_*k*_ + *f*_*cl*_ + *f*_*m*_ = 1); and ${K}_{e}^{\ast }$ and ${{\boldsymbol{\mu }}}_{e}^{\ast }$ are the calculated effective bulk and shear moduli, respectively.
6. Elastic anisotropy results from the laminated clay and laminated kerogen. The effective elastic modulus of laminated clay and laminated kerogen was calculated using the Backus averaging model. The calculated output represents the anisotropic effective elastic properties of shale rocks.
7. The P-wave velocities in the effective anisotropic medium can be written as follows:

$${V}_{PV}=\sqrt{\frac{{C}_{33}^{\ast }}{\rho }}$$

where ρ is the average density; and V_PV_ is for the vertically propagating P-wave, which is the measured velocity in acoustic logging. Compressional slowness is the reciprocal of the P-wave velocity.

## Application

**Figure 2:**
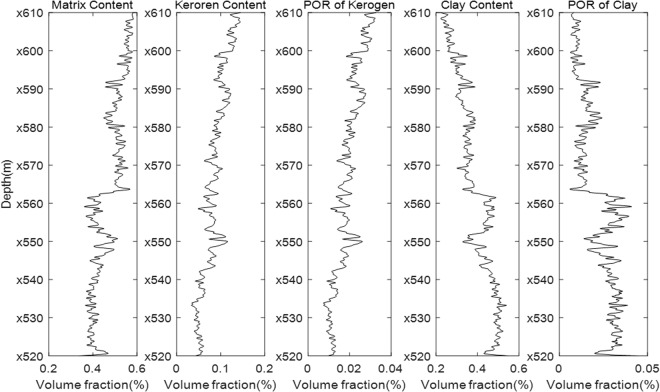
Well logging data of the rock composition in a shale reservoir.

**Figure 3:**
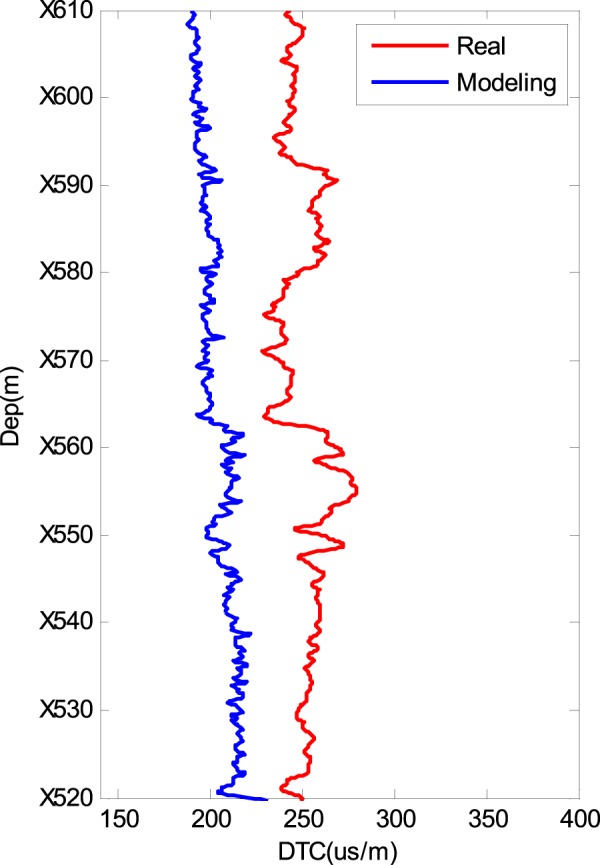
Modelling results using an isotropic shale rock physics model.

**Figure 4:**
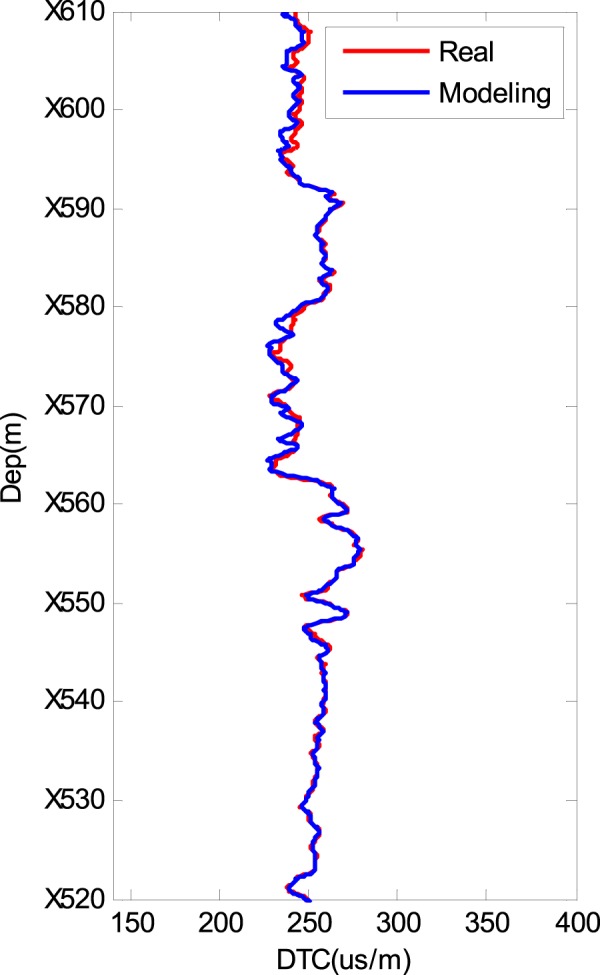
Modelling results of the real well logging data shown in Fig. 1.

Figure 2 shows the rock composition data of a real well in a shale gas reservoir in China that were obtained using well logging instruments. The composites of the matrix in this well are calcite, quartz and dolomite. POR indicates porosity. Because we do not have the volume fraction of the layered clay and layered kerogen in the rock composition data, we first simulate the DTC result using our modelling procedure with an isotropic model. The modelling results are shown in Fig. 3. The modelled DTC (blue line) is approximately 210 µs/m, whereas the real DTC is approximately 250 µs/m. In addition, the real DTC changes more substantially than the modelled DTC. Although the general trends of the two lines are similar, this cannot be an acceptable modelling result. Further observations of the composition data show that the volume fractions of the layered clay and layered kerogen are unknown. However, the influences on the DTC of the layered clay and layered kerogen are very large. Therefore, we change the volume fractions (ratio of layer clay/kerogen volume to total clay/kerogen volume) of the layered clay and layered kerogen and determine the correct volume fractions of the two parts from 0 to 100% to obtain the most consistent DTC modelling results for certain layered clay and layered kerogen volume fractions. Figure 4 shows the shale rock physics modelling results using our proposed simulation procedure. For comparison, the measured compressional slowness (DTC) is plotted in Fig. 4 as a red line. The modelling result is plotted as a blue line. The two lines are consistent with each other, which shows the efficiency of our shale rock physics modelling procedure.

**Figure 5:**
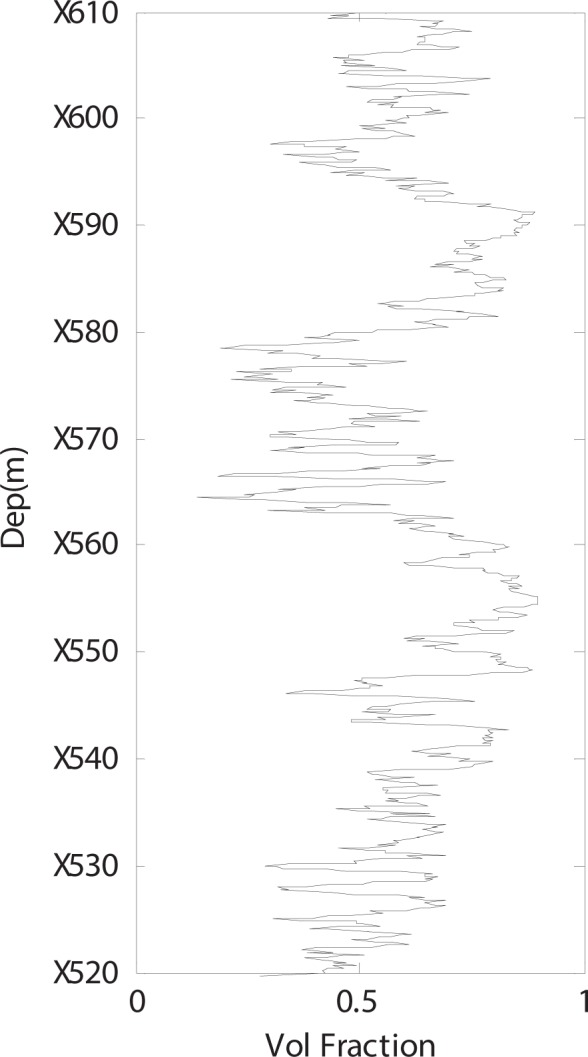
Volume fraction of layered kerogen with respect to the total kerogen volume.

**Figure 6:**
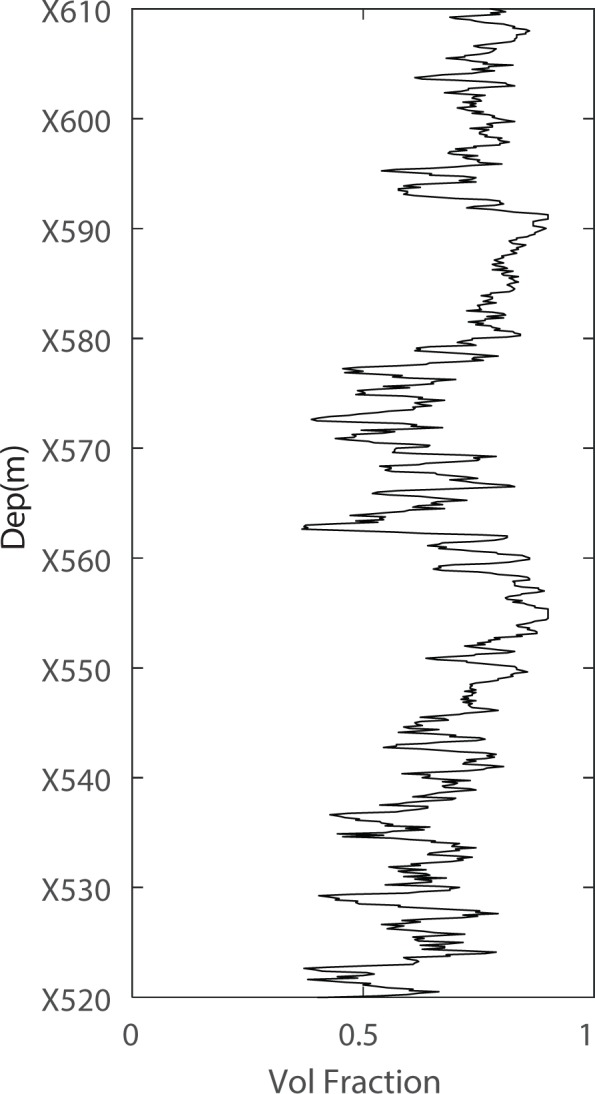
Volume fraction of layered clay with respect to the total clay volume.

As shown in Fig. 4, a satisfactory modelling result is observed. However, certain issues should be discussed. Figure 5 shows the volume fraction of layered kerogen used to perform the simulation, and Fig. 6 shows the volume fraction of layered clay used to perform the simulation. After we add the layered clay and layered kerogen, the satisfactory results shown in Fig. 4 are obtained. This adjustment is also a confirmation that our modelling procedure is efficient for shale rock physics modelling.

## Conclusions

Due to the different composites and properties of shale rock, we propose a modelling procedure that uses an appropriate modelling method for different compositions of shale rock. The frequency range of acoustic logging measurements represents an important issue for choosing efficient modelling methods. In the logging frequency range, compressional and shear velocity (slowness) can be calculated directly using elastic parameters. Laminated clay and kerogen have significant effects on the vertical velocity, which can be measured by acoustic logging. By setting certain laminated clay and kerogen volume fractions, we can obtain consistent results between the modelled DTC and measured DTC, which verifies our shale rock physics modelling procedure.
